# The Effect of Green Tea Extract on Reproductive Improvement in Estradiol Valerate-Induced Polycystic Ovarian Syndrome in Rat

**Published:** 2015

**Authors:** Habibeh Ghafurniyan, Mahnaz Azarnia, Mohammad Nabiuni, Latifeh Karimzadeh

**Affiliations:** *Department of Cell and Molecular Biology, School of Biological Sciences, Kharazmi University, Tehran, Iran.*

**Keywords:** Polycystic ovary syndrome, Wistar rat, Green tea extract, Insulin resistance

## Abstract

Polycystic ovary syndrome (PCOS) is a reproductive and metabolic disorder in which the level of oxidative elements in blood rises. Green tea is a potent antioxidant since it contains catechins. In this study, the effect of hydro-alcoholic green tea extract on PCOS rats was examined. For PCOS induction, 96 mature Wistar rats were given estradiol valerate. After 60 days, the rats were divided into four groups including PCOS group and three experimental groups, which were given 50, 100 and 200 mg/Kg BW green tea extract 10 days, intraperitoneally. The serum concentration level of FSH, LH, testosterone and insulin were measured using ELISA method, while the serum concentration level of glucose was measured using glucose oxidase methods. Insulin resistance was calculated using HOMA-IR formulation. The data were analyzed using the one-Way ANOVA method considering P<0/05 level of significance.

There were a significant reduction in LH serum level, body and ovarian weight between the green tea extract treated-groups compare to PCOS. Moreover, a significant reduction in insulin resistance index was seen in the treatment groups related to PCOS. *Histomorphometric studies* also showed the significant changes in the number of follicles and theca layer thickness.

These changes demonstrated a marked improvement in the symptoms of PCOS which may be due to green tea effects on oxidative stress pathways. Green tea can be considered as a potentially effective drug for treatment of PCOS, Insulin resistance and Type II diabetes.

## Introduction

Polycystic ovary syndrome (PCOS) is a reproductive hormonal abnormality and a metabolic disorder. This syndrome affects an estimated 5–10% of reproductive-age women. This is the main cause of infertility (1, 2). Chronic anovulation is common in women with PCOS; however, spontaneous ovulation and pregnancy may occur occasionally in women with PCOS. Menstrual irregularity is considered as one of the symptoms of PCOS. This irregularity usually continues after menarche; as a result, women with PCOS may not have regular menstrual patterns (2).

Classical morphology of PCOS includes ovarian cortical thickening, multiple tiny capsular follicular cysts, hyperplasia, luteinized inner theca, stromal hyperplasia and multiple immature follicles, which is an indication of cessation of folliculogenesis. The size of ovary ranges from normal size to a very large size (the volume of the ovary increases to more than 10 cm). In ultrasonography, histologic findings appear as a peripheral ring of at least eight small follicles (6-10 mm diameter) (2, 3). Biochemical abnormalities of PCOS are characterized as: increased serum concentrations of androgenic hormones such as testosterone and androstenedione, Dehydroandrostenedione, Dehydroandrostenedione sulfate, 17 – hydroxyprogesterone (4). Moreover, hirsutism, oligomenorrhea, amenorrhea (5, 6), impaired release of gonadotropin associated with increased secretion of LH compared to FSH are observed in these patients (2). Such disorders as visceral obesity and increased body fat and diabetes mellitus type II; cardiovascular abnormalities seem to be common in PCOS. Moreover, this disease is associated with insulin resistance, increased insulin level and drop in blood glucose level (7).

Green tea is produced from the leaves and buds of *Camellia sinensis*. It is almost the most widely consumed beverage in the world. Green tea is a non-fermented tea (8) and contains a large amount of catechins. Catechins are potent antioxidants, both *in-vitro* and *in-vivo*. In addition, green tea contains some minerals and vitamins, which increase the antioxidant potential of this kind of tea (9). Recent studies on humans show that green tea has many health benefits including reduced risk of cardiovascular disease and some cancers, anti-effects on blood pressure, body weight control (weight loss), antivirus and antibacterial activities, involving in anti-diabetic processes (through saliva and intestinal amylase inhibitor that prevents the breakdown of starch). It also has anti-mutagenic, anti-inflammatory, and anti-fibrotic properties. It also protects against ultraviolet radiation, increases bone mineral density, and reduces cholesterol and triglycerides (10) and decrease insulin resistance. Certain studies indicate that green tea can effectively affect obesity. It also causes anti-insulin resistance, anti-diabetic effects (11). All these cases are the metabolic results of polycystic ovary syndrome (6, 7 and 9). Hence, the effect of green tea on the polycystic ovary was examined in this study. 

## Experimental

*Methods*

The green tea was collected in Lahijan city located in Gilan province. The collected samples were dried in the shade and its leaves were removed from the plants’ stems. Then, they were pulverized to a powder form by mechanical means. To prepare hydro-alcoholic extract, 200 g of the powder of the dried green tea leaves were soaked with 1500 mL of ethyl alcohol 80% for 48 hours. The obtained solution was evaporated after it was filtrated in the constant flow of the air. Then, it was completely dried. Thereafter, dried extract was collected. Then, the hydro-alcoholic extract of the green tea was used to prepare the desired dose of the treatment (12).

This study was conducted on 96 Adult female Wistar rats weighing 200 ± 20 gram (7–8 wk of age) from the animal house of the Kharazmi University, Tehran, Iran. Animals were maintained kept in a central animal care facility, housed in plastic cages (30- 19- 13 cm) under a 12-h light, 12-h dark cycle (Lights on from 08:00 to 20:00), Humidity and temperature were set at 55±15% and 20-24 ˚C, respectively, and a free access to water and commercial food (Behparvar Com., Iran). The rats under study were maintained for at least 7 days in the above-mentioned conditions before drug administration, so that they can completely get accustomed to the environment. All procedures were carried out according to the Guide for the Care and Use of Laboratory Animals (National Research Council 1996).

The selected animals for the case of induction of polycystic ovary have a minimum of two consecutive regular estrous cycles after providing daily vaginal smear tests for 14-12 days. Rats were in the estrous stage of their reproduction cycle (2). The method used in this project was hormonal induction using estradiol valerate. In order to conduct this procedure, rats were given daily subcutaneous injections of 2 mg/Kg estradiol valerate (Aburaihan Co. Iran) once a day. Then, daily vaginal smear test was made. This process continued until variations in estrous cycle as well as irregular estrous cycles were observed. It should be noted that this process also continued until the onset of the advance of the Persistent Vaginal Cornification (PVC) step was seen. This was usually observed 60 days after the injection of estradiol valerate (13). In order to confirm the complete induction of PCOS Serological tests via radioimmunoassay method and histomorphometric studies by the hematoxylin and eosin technique was performed. Ovaries were placed in bouin fixative for histological analysis; blocked Samples were sliced to sections of 7 micron thickness using a microtome to staining with hematoxylin-eosin for histological observations. In order to determine follicular development, follicles were classified based on morphology and diameter into 6 groups of primordial, primary, preantral (all with <600 µm in diameter), antral (with 600-1000 µm in diameter), cystic follicles and corpus luteum.

If the syndrome was induced, the LH level would increase while FSH level would decrease. Moreover, histomorphometric studies indicated an increase in the number of primordial, primary, pre-antral, antral follicles and corpus luteum. It also showed a decrease in the number of cystic follicles. 

The rest of the induced rats is divided into five experimental groups: PCOS rats that received no injection (PCOS-control or PCOS), The rats that received saline (PCOS-sham), PCOS rats that were injected with 50 mg/Kg BW green tea extract intraperitoneally (GT-treated-1), PCOS rats that were injected with 100 mg/Kg BW green tea extract intraperitoneally (GT-treated-2), PCOS rats that were injected with 200 mg/Kg BW green tea extract intraperitoneally (GT-treated-3). At the end of 10 days period, the serum concentration level of FSH and LH and testosterone were measured using by Rat/mouse ELISA Kit (Cosmo Bio Co. *Ltd.* Japan), while the serum concentration level of insulin and glucose were measured using respectively Insulin Rat Ultrasensitive ELISA (ALPCO Diagnostics, USA) and glucose oxidase reaction (Glucose Oxidase Analyzer, Beckman, Fullerton, CA). Histomorphometric studies were performed by the hematoxylin and eosin technique as described above.  Homeostasis Model Assessment- insulin resistance (HOMA-IR) was calculated using the formula, as described by Matthews *et al.* (14): (fasting insulin [mU/L] ×fasting glucose [mmol/L]/22.5). PCOS-sham group was later removed from the experiment due to its lack of any difference with the PCOS-control group.

*Analysis of data*

Hormonal and histological results obtained from the studies using the rats in all groups were compared by SPSS software and One - Way ANOVA. The results were expressed as mean ± standard error (Mean ±SEM). The level of significance was considered as P <0.05.

## Results and Discussion

*Hormonal findings*

Levels of LH and testosterone in PCOS group were increased while the level of FSH was decreased compared to the control group. In the groups treated with different doses of green tea for 10 days, it was observed that injection of green tea resulted in significant decrease in the level of LH and testosterone and no significant change in FSH in all three experimental groups compared to the PCOS control group (Table 1). 

**Figure 1:**
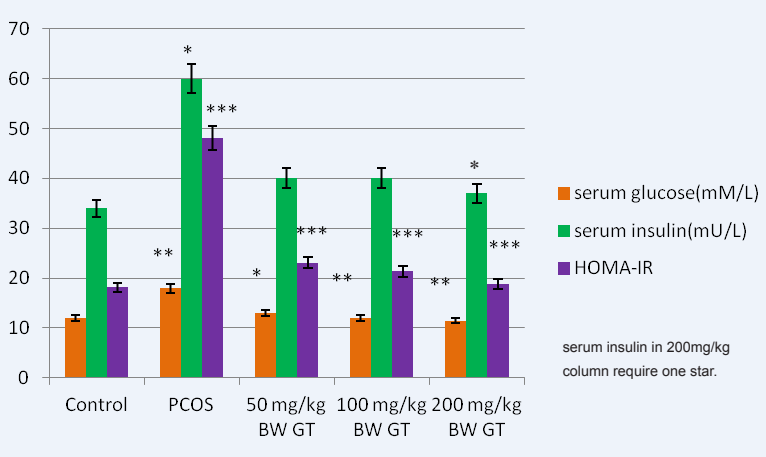
Comparison of the glucose, insulin and HOMA-calculated insulin resistance levels in control, PCOS and experimental groups. Relative to PCOS group, a significant decrease in the glucose relative to PCOS group, a significant decrease in the glucose, insulin (in 200mg/kg BW GT group) and HOMA- calculated insulin resistance level is seen in all green tea extract-treated groups, interaperitoneally. and HOMA-calculated insulin resistance level is seen in green tea extract-treated groups, intraperitoneally.GT: Green Tea, HOMA-IR: Homeostasis Model Assessment- insulinresistance. ***P<0.001; **P<0.01; *P<0.05.

The results suggest that green tea reduces the amount of glucose in the experimental group compared to the PCOS control group. In addition, a non-significant decrease in the level of insulin in the treatment groups was observed. A significant decrease in the HOMA-calculated insulin resistance was observed at green tea extract-treated groups in a *dose*-*dependent manner* (Figure 1).

*Histomorphometric findings*

Examining the changes in the body weight in the experimental group compared to the PCOS control group showed that green tea extract reduced body weight in the experimental groups (Table 1).

The results obtained from changes in the ovarian weight showed that ovarian weight increased after induction of polycystic ovary syndrome. Then, green tea significantly reduced the large size of the polycystic ovaries compared to the PCOS control group ovaries (Table 1). 

**Table 1:** Comparison of the mean levels of LH, testosterone, body and ovarian weight of the rats in the control, PCOS and experimental groups. ***P<0.001; **P<0.01; *P<0.05

**Groups **	**LH**	**Testosterone(ng/dl)**	**Body Weight(g)**	**Ovarian Weight(mg)**
**Control **	2.8±0.01	50±3.30	163±08	9.71±0.12
**PCOS**	6.75±0.05 (***)	113±50.15 (***)	222.8±36.8 (***)	15.02±0.17 (***)
**50 mg/Kg BW GT**	2.22±0.09 (**)	101±15.11	207±15 (*)	12.4±0.3 (*)
**100 mg/Kg BW GT**	2.19±0.06 (***)	96±40 (*)	196±12 (**)	11.23±0.62 (**)
**200 mg/Kg BW GT**	1.92±0.02 (***)	93.5±40 (*)	194±11 (***)	11.33±0.54 (**)

**Figure 2:**
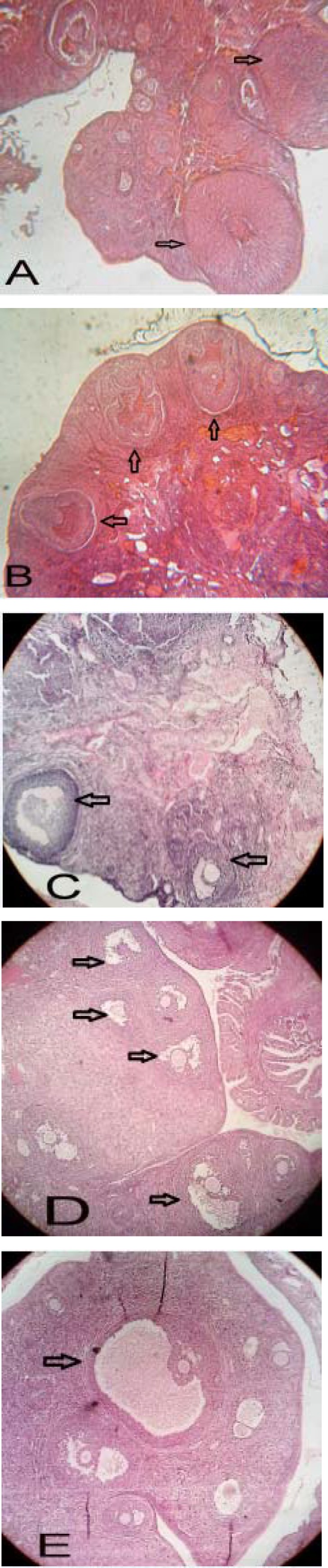
Histological analysis of normal (A), PCOS (B) and 50, 100 and 200 mg/Kg BW green tea extract -treated PCOS (C, D and E respectively) ovaries. Arrows indicate the corpus luteum in figure A, some cysts in figure B, some antral follicles in figure C, some late pre antral follicles in figure D and *Gratian* follicles in figure E. Ovarian sections were stained with hematoxylin and eosin, X400.

Examining morphology of the tissue sections prepared from ovaries in the experimental group of follicles at different stages including graph showed that the follicles had thick granular layer and thin theca compared to the PCOS control group. Moreover, a significant number of corpus luteum, indicators of ovulation, was observed. However, greater number of cystic follicles was observed in the PCOS control group. They had thin granular layer and thick theca. Moreover, low number of corpus luteum was observed which indicated that injection of estradiol valerate induced polycystic ovaries reduced the number of active follicles as well as ovulation (Figure 2).

 Morphological studies of the treatment groups showed that injection of hydro-alcoholic green tea extract in 10 days increased the number of follicles at various stages. It also increased the number of corpus luteum and reduced the number of cystic follicles (Figure 2). 

Injection of green tea extract at all three doses of 50 mg/Kg, 100 mg/Kg and 200 mg/Kg showed significant changes in ovarian tissues compared with the control group. Counting the number of follicles in various stages showed that the injection of estradiol valerate reduced the number of PAF, AF, CL and PF follicles; however, it increased the number of cystic follicles compared to control group. This reduction is significant. However, in the groups that received the extract (experimental group), the number of these follicles significantly increased while the number of cystic follicles significantly decreased. It is noteworthy that if doses of green tea increases, the observed changes will be more significant (Table 2). 

**Table 2:** Comparison of the number of Different follicular groups in control and PCOS and green tea extract-treated ovaries (n=10). Note the increase in the numbers of cysts and a decrease in the number of corpus luteum of PCOS ovaries, respectively. A significant increase in all follicular clusters is detected in green tea extract -treated PCOS ovaries. Moreover, a significant decrease in the number of ovarian cysts is also detected. PMF, Primordial Follicle; PF, Primary Follicle; PAF, Preantral Follicle, AF, Antral Follicle; CF, Cystic Follicle; CL, Corpus Luteum. ***P<0.001; **P<0.01; *P<0.05

**Groups **	**The number of primordial follicles**	**The number of Primary Follicles**	**The number of Preantral follicles**	**The number of Preantral follicles**	**The number of Cystic Follicles **	**The number of corpus luteum**
**Control **	**45.5±0.33**	**20.57**	**28.66±0.33**	**18.66±0.33**	**0**	**10.33±.36**
**PCOS**	41±0.50(*)	9.66±0.03 (***)	4.66±0.02 (***)	2.66±0.33 (***)	4.66±0.13 (***)	3.33±0.23 (***)
**50 mg/Kg BW GT**	**41±0.52 **	16.33±0.05 (***)	18.66±0.06 (***)	18.66±0.33 (***)	1.66±0.06 (***)	9.66±0.33 (***)
**100 mg/Kg BW GT**	**42±0.57 **	18.6±0.06 (***)	25.66±0.09 (***)	25.66±0.66 (***)	0.33±0.01 (***)	8.33±0.31 (***)
**200 mg/Kg BW GT**	**43±0.61 **	20.6±0.07 (***)	31.33±0.18 (***)	31.33±0.88 (***)	0 (***)	9.01±0.38 (***)

**Figure 3:**
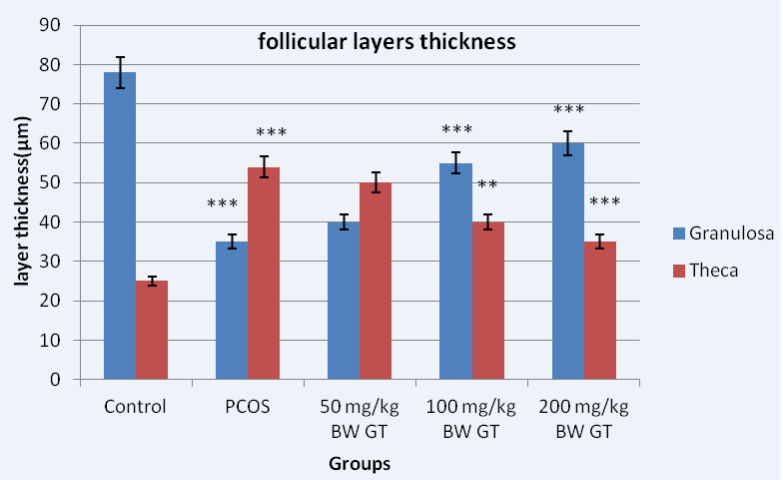
Diameter of the granular and theca layers thickness in control, PCOS control and experimental groups. Measuring the thickness of follicular layers showed the thickness of the theca layer in the PCOS control group significantly increased compared to the control group. However, the thickness of the theca layer in the experimental groups significantly decreased compared to the PCOS control group. Relative to PCOS control group, *10-day* sequential treatment with the green tea extract resulted in a significant increase in theca and a significant decrease in granulosa layer thickness. ***P<0.001; **P<0.01.

Measuring the diameter of the graph follicles in PCOS control group compared to the graph showed significant increase in diameter of the follicles; however, the diameter of the follicles significantly decreased in the experimental group compared to PCOS control group. The thickness of the granular layer in the PCOS control group significantly decreased compared to the control group while the thickness of the granulosa layer in the PCOS control group significantly increased compared the experimental group (Figure 3). 

Measuring the thickness of the theca layer showed reverse result compared to the results obtained from measuring the thickness of the granulosa layer. Thus, the thickness of the theca layer in the PCOS control group significantly increased compared to the control group. However, the thickness of the theca layer in the experimental groups significantly decreased compared to the PCOS control group (Figure 3).

## Discussion

 Oxidative stress refers to the imbalance between the production of the free radicals of oxygen and antioxidant defense capacity of the body. Naturally, reactive oxygen species are naturally generated during the body’s metabolic reactions. They can react with important macromolecules of the body such as lipids, proteins and nucleic acids. In normal conditions, there is a balance between both production and elimination. The imbalance between these two processes can lead to oxidative stress and pathological changes in different cells. ROS can cause diseases such as cancer, cardiovascular diseases, diabetic nephropathy and aging (15).

In this study, significant increase in the level of glucose and insulin can be observed. This disorder may refer to insulin resistance, which can be attributed to oxidative stress process. In PCOS, the generated free radicals of the oxygen species may be stimulated by mononuclear cell which is caused by hyperglycemia and obesity (16). Therefore, toll like receptors may cause insulin resistance, hyperandrogenism, development of atherosclerotic lesions, decreased oocyte maturation and follicular development in the ovary in PCOS condition (14, 17 and 18). 

Christopher studies showed that insulin directly stimulates the granular layer cells of the ovaries to produce estradiol. It also stimulates the theca cells to produce androgens. Production of androgens by theca cells stimulates LH by increasing the aromatizing activity in the FSH as well as granular cells (19). In this study, the increased levels of androgens like testosterone as well as insulin resistance were observed. High insulin levels increase the androgen levels in three ways: 1 – affecting the insulin receptor which increases androgen response of theca cells to LH. 2 – Reducing production of SHBG in the liver. 3 - Lowering production of the proteins which bind to IGF (20). The results obtained from the present study showed that serum insulin level in the experimental group reduced after 10 days infusion of green tea extract. This decrease was significant only at 200 mg/Kg dose of green tea extract. This result is line with those obtained from Lim researches (21).

Moreover, the increase observed in the thickness of the fatty tissue in this experimental model play an important role in the development of insulin resistance as well as its regulation by secretion of adiponectin. Based on previous findings, green tea increases secretion of adiponectin from adipocytes. The occurrence of Type II diabetes may due to a defect in the proper differentiation of adipocytes by adiponectin (13, 22 and 23). Bhathena and Velasquez demonstrated the potential role of green tea in controlling gain weight. They also showed that weight loss improves the fertility variables in PCOS obese women. Weight loss not only has a direct effect on the frequency of ovulation, but also increases the possibility that the patients with PCOS respond to infertility treatment drugs such as clomiphene citrate and gonadotropins (22, 23). Hence, the effects of green tea on weight loss in rats and its subsequent effects on adjusting the reproductive factors were examined in this study. The results obtained from this study confirmed the results obtained from previous studies that said Catechins found in green tea directly can connect to the peroxisome proliferator-activated receptors (PPARs), regulates adipocyte differentiation and expression of adiponectin (21). These data are in line with our results that showed reduced insulin resistance and reduced fasting glucose. 

Calabro *et al.* showed that after induction of PCOS, follicle growth and development in the experimental group decreased compared to the control group. These results are consistent with the findings of the present study (24). Moreover, Desjardins *et al.* reported that multiple cysts were generated in the ovaries after induction of PCOS in rats. The origin of these cysts was atretic follicles. They also observed that granulosa cells were degenerated; moreover, the outer layer of the theca calls were thickened (25). We show for the first time that green tea extract causes a decrease in the thickness of the follicular theca layer in PCOS rats, possibly mediated through increased lipolysis and decreased hypertrophy of this layer. Due to this decrease, the androgens and steroids produced by this layer would also decrease. The results of the present study also demonstrated decreased number of follicles, corpus luteum as well as appearance of the cysts in the ovary. Green tea extract increases the number of follicles and corpus luteum while it decreases the number of cystic follicles in the ovary. 

PCOS is associated with changes in sex hormones, especially steroid. However, in polycystic group, sensitivity of the pituitary and hypothalamus changed due to increased level of estrogen (26, 27). The effect of 10-day infusion of green tea extract on hormonal levels showed that lutenizing hormonal levels decreased significantly. As a result, consistent high levels of LH as well as constant low FSH levels were observed. These findings were in line with those obtained by some researchers (28, 29 and 30). Green tea consumption can regulate secretion of hormones. It also affects the hormones’ receptors and reduces the adverse effect of hormone imbalance. High levels of FSH in the normal control samples resulted in follicular growth and development; moreover, phasic LH release caused induction of ovulation as well as generation of corpus luteum. 

## Conclusion

Green tea consumption causes modulating gonadotropin levels, reducing insulin resistance, losing rats weights and improving the ovarian morphology. Due to these systemic effects and the ability to reduce metabolic features, Green tea has been able to increase the reproduction rate in PCOS rats through a reduction in ovarian cysts and an increase in the appearance of corpus luteum. Therefore, Green tea can alter both reproductive and the metabolic features of PCOS. Furthermore, we speculate that this effect of Green tea is mediated through glucose-insulin signaling pathway. It is suggested that green tea is more effective in high concentrations. 

## References

[1] Hassanzadeh Bashtian M, Emami SA, Mousavifar N, Esmaily HA, Mahmoudi M, Mohammadpoor AH (2013). Effects Seeds Extract on Insullin Resistance in Women with Polycystic Ovarian Syndrome. Iran. J. Pharm. Res..

[2] Ehrmann DA (2005). Polycystic ovary syndrome. New England J. Med..

[3] Atiomo WU, El-Mahdi E, Hardiman P (2003). Familial associations in women with polycystic ovary syndrome. Fertility and Sterility.

[4] Sabuncu T, Harma M, Harma M, Nazligul Y, Kilic F (2003). Sibutramine has a positive effect on clinical and metabolic parameters in obese patients with polycystic ovary syndrome. Fertility and Sterili.

[5] Karimzadeh L, Nabiuni M, Sheikholeslami A, Irian S (2012). Bee venom treatment reduced C-reactive protein and improved follicle quality in a rat model of estradiol valerate-induced polycystic ovarian syndrome. J. Venomous Animals and Toxins Including Tropical Dis..

[6] Teixeira Filho FL, Baracat EC, Lee TH, Suh CS, Matsui M, Chang RJ, Shimasaki S, Erickson GF (2002). Aberrant expression of growth differentiation factor-9 in oocytes of women with polycystic ovary syndrome. J. Clinical Endocrinol. Metabolism.

[7] Hunter MH, Sterrett JJ (2000). Polycystic ovary syndrome: it's not just infertility. American Family Physician.

[8] Yildirim B, Sabir N, Kaleli B (2003). Relation of intra-abdominal fat distribution to metabolic disorders in nonobese patients with polycystic ovary syndrome. Fertil. Steril..

[9] Haidari F, Omidian K, Rafiei H, Zarei M, Mohamad Shahi M (2013). Green Tea (Camellia Sinesis) Supplementation to Diabetic Rats Improves Serum and Hepatic Oxidative Stress Markers. Iran. J. Pharm. Res..

[10] Mann J, Truswell S (2012). Essentials of human nutrition. Oxford Univ. Press.

[11] Sabu MC, Kuttan R (2002). Anti-diabetic activity of medicinal plants and its relationship with their antioxidant property. J. Ethnopharmacol..

[12] Raatz SK, Torkelson CJ, Redmon JB, Reck KP, Kwong CA, Swanson JE, Liu C, Thomas W, Bantle JP (2005). Reduced glycemic index and glycemic load diets do not increase the effects of energy restriction on weight loss and insulin sensitivity in obese men and women. J. Nutrition.

[13] Karimzadeh L, Nabiuni M, Kouchesfehani HM, Adham H, Bagheri A, Sheikholeslami A (2013). Effect of bee venom on IL-6, COX-2 and VEGF levels in polycystic ovarian syndrome induced in Wistar rats by estradiol valerate. J. Venomous Animals and Toxins Including Tropical Dis..

[14] Matthews DR, Hosker JP, Rudenski AS, Naylor BA, Treacher DF, Turner RC (1985). Homeostasis model assessment: insulin resistance and Î²-cell function from fasting plasma glucose and insulin concentrations in man. Diabetologia.

[15] Evans JL, Goldfine ID, Maddux BA, Grodsky GM (2002). Oxidative stress and stress-activated signaling pathways: a unifying hypothesis of type 2 diabetes. Endocrine Rev..

[16] Jain SK (1989). Hyperglycemia can cause membrane lipid peroxidation and osmotic fragility in human red blood cells. J. Biol. Chem..

[17] Chattopadhayay R, Ganesh A, Samanta J, Jana SK, Chakravarty BN, Chaudhury K (2009). Effect of follicular fluid oxidative stress on meiotic spindle formation in infertile women with polycystic ovarian syndrome. Gynecol. Obstetric Investigat..

[18] Dandona P, Thusu K, Cook S, Snyder B, Makowski J, Armstrong D, Nicotera T (1996). Oxidative damage to DNA in diabetes mellitus. The Lancet.

[19] Tymchuk CN, Tessler SB, Barnard RJ (2000). Changes in sex hormone-binding globulin, insulin, andserum lipids in postmenopausal women on alow-fat, high-fiber diet combined with exercise. Nutrition Cancer.

[20] Namita P, Mukesh R, Vijay KJ (2012). Camellia Sinensis (green tea): A review. GJP.

[21] Lim DY, Lee ES, Park HG, Kim BC, Hong SP, Lee EB (2003). Comparison of green tea extract and epigallocatechin gallate on blood pressure and contractile responses of vascular smooth muscle of rats. Archives of Pharmacal. Res..

[22] Bhathena SJ, Velasquez MT (2002). Beneficial role of dietary phytoestrogens in obesity and diabetes. The American J. Clinical Nutrition.

[23] Moran LJ, Noakes M, Clifton PM, Wittert GA, Williams G, Norman RJ (2006). Short-term meal replacements followed by dietary macronutrient restriction enhance weight loss in polycystic ovary syndrome. The American J. Clinical Nutrition.

[24] Calabro P, Willerson JT, Yeh ETH (2006). Inflammatory cytokines stimulated C-reactive protein production by human coronary artery smooth muscle cells. Circulation.

[25] Desjardins GC, Beaudet A, Brawer JR (1990). Alterations in opioid parameters in the hypothalamus of rats with estradiol-induced polycystic ovarian disease. Endocrinol..

[26] Balen AH, Conway GS, Kaltsas G, Techatraisak K, Manning PJ, West C, Jacobs HS (1995). Andrology: Polycystic ovary syndrome: the spectrum of the disorder in 1741 patients. Human Reproduct..

[27] Vigorito C, Giallauria F, Palomba S, Cascella T, Manguso F, Lucci R, De Lorenzo A, Tafuri D, Lombardi G, Colao A (2007). Beneficial effects of a three-month structured exercise training program on cardiopulmonary functional capacity in young women with polycystic ovary syndrome. J. Clinical Endocrinol. Metabolism.

[28] Orio F, Giallauria F, Palomba S, Manguso F, Orio M, Tafuri D, Lombardi G, Carmina E, Colao A, Vigorito C (2008). Metabolic and cardiopulmonary effects of detraining after a structured exercise training programme in young PCOS women. Clinical Endocrinol..

[29] Palomba S, Giallauria F, Falbo A, Russo T, Oppedisano R, Tolino A, Colao A, Vigorito C, Zullo F, Orio F (2008). Structured exercise training programme versus hypocaloric hyperproteic diet in obese polycystic ovary syndrome patients with anovulatory infertility: a 24-week pilot study. Human Reproduct..

[30] Chen MP, Chung FM, Chang DM, Tsai JCR, Huang HF, Shin SJ, Lee YJ (2006). Elevated plasma level of visfatin/pre-B cell colony-enhancing factor in patients with type 2 diabetes mellitus. J. Clinical Endocrinol. Metabolism.

